# Breaking the sleep tunnel: effects of a single-session scarcity-informed mass psychoeducation on dysfunctional beliefs and insomnia symptoms

**DOI:** 10.1093/sleepadvances/zpag099

**Published:** 2026-09-03

**Authors:** Chang-Ho Yun, Saeil Nam, Hyeongsuk Ryu, Sang-Min Paik, Jae Rim Kim, Jee-Eun Yoon

**Affiliations:** Department of Neurology, Seoul National University Bundang Hospital and Seoul National University College of Medicine, Seongnam, Republic of Korea; Department of Neurology, Seoul National University Bundang Hospital and Seoul National University College of Medicine, Seongnam, Republic of Korea; Department of Neurology, Seoul National University Bundang Hospital and Seoul National University College of Medicine, Seongnam, Republic of Korea; Department of Neurology, Seoul National University Bundang Hospital and Seoul National University College of Medicine, Seongnam, Republic of Korea; Department of Neurology, Chungnam National University Sejong Hospital, Chungnam National University School of Medicine, Sejong, Republic of Korea; Department of Neurology, Soonchunhyang University Bucheon Hospital, Bucheon, Republic of Korea

**Keywords:** insomnia, psychoeducation, dysfunctional beliefs about sleep, cognitive-behavioral therapy for insomnia, scarcity theory, single-session intervention, mass intervention, minimal clinically important difference

## Abstract

**Study Objectives:**

Cognitive-behavioral therapy for insomnia (CBT-I) effectively targets dysfunctional sleep beliefs but remains inaccessible to most. This study examined whether a single 60-minute mass psychoeducation session, incorporating Scarcity Theory with CBT-I psychoeducation elements and Acceptance and Commitment Therapy concepts, could reduce dysfunctional beliefs and insomnia symptoms over 4 weeks.

**Materials and Methods:**

This prospective, single-arm study was conducted at Seoul National University Bundang Hospital. Of 182 lecture attendees, 81 consented, 40 completed baseline and immediate post-lecture assessments, and 29 completed all four timepoints through 4 weeks. The primary outcome was the Korean Dysfunctional Beliefs and Attitudes about Sleep-6 (K-DBAS-6). Secondary outcomes included the Insomnia Severity Index-3 (ISI-3), Bandwidth Tax Visual Analogue Scale (BT-VAS), and Patient Global Impression measures. Longitudinal effects were analyzed using repeated measures ANOVA (*N* = 29).

**Results:**

K-DBAS-6 decreased significantly (*d* z = 0.63, p = .003). ISI-3 (*d* z = 0.60, p = .003), BT-VAS (*d* z = 0.62, p = .002), and PGI-S (*d* z = 0.97, p < .001) also improved. BT-VAS showed immediate post-lecture reduction (*d* z = 0.63, p < .001; *N* = 40). At 4 weeks, 72.4% reported global improvement (PGI-I ≤ 3). Anchor-based minimal clinically important differences were 1.32 (K-DBAS-6), 1.58 (ISI-3), and 1.83 (BT-VAS). All effects remained significant after adjusting for age and sex.

**Conclusions:**

A single mass psychoeducation session informed by Scarcity Theory produced significant, sustained reductions in dysfunctional sleep beliefs and insomnia symptoms with medium effect sizes. This scalable approach warrants investigation as a first-step intervention for insomnia.

## Teaser

Most people with insomnia never receive effective treatment because standard therapies require multiple sessions with trained specialists. This study tested whether a single 1-hour public lecture could make a difference. Using the concept of a “bandwidth tax” from behavioral economics, the lecture helped attendees to understand how worry about sleep drains mental resources and perpetuates poor sleep. Participants showed meaningful improvements in sleep-related beliefs and insomnia symptoms lasting at least 4 weeks, with nearly three-quarters reporting overall improvement. Because the lecture is freely available online, it can reach far more people than conventional treatments. These findings suggest that brief, accessible educational interventions may help to address the global insomnia treatment gap.

## Graphical abstract

**Graphical Abstract:**
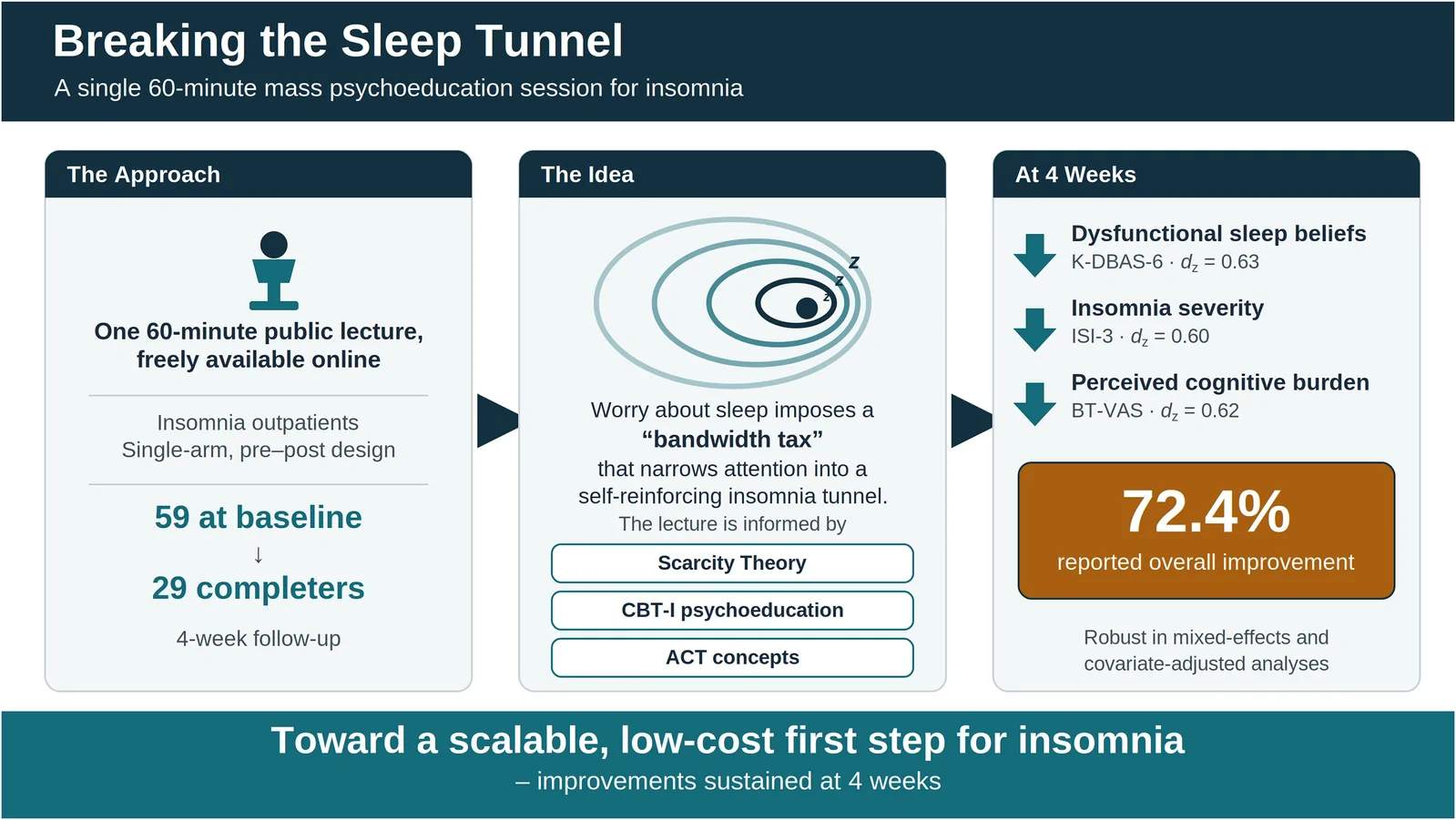

## Introduction

Insomnia is among the most prevalent sleep disorders, with approximately one-third of the general adult population reporting insomnia symptoms and 6%–13% meeting diagnostic criteria for insomnia disorder [1,2]. Beyond its immediate impact on sleep quality and daytime functioning, insomnia is increasingly recognized as a transdiagnostic risk factor for mental health disorders. Longitudinal meta-analytic evidence demonstrates that insomnia confers a twofold increase in the risk of developing depression, and elevated risk for anxiety disorders, substance use disorders, and psychosis [3–5]. The persistence of untreated insomnia symptoms and their potential to progress to chronic insomnia disorder underscore the importance of early, accessible intervention [6]. Cognitive-behavioral therapy for insomnia (CBT-I) is the recommended first-line treatment for chronic insomnia, with robust evidence supporting its efficacy across systematic reviews, meta-analyses, and clinical guidelines [7–10].

Despite its established efficacy, CBT-I remains largely inaccessible. The treatment requires trained specialists, typically involves [4–8] sessions, and is unavailable in most primary care and community settings [11]. This accessibility gap has motivated the development of alternative delivery formats. Digital CBT-I (dCBT-I), delivered through web platforms and mobile applications, has demonstrated medium-to-large effects on insomnia severity comparable to face-to-face delivery [12–14]. Guided and unguided self-help interventions have also shown promise [15]. A stepped-care model has been proposed in which brief, scalable interventions serve as the first step, with more intensive treatments reserved for non-responders [11]. However, even digital and self-help formats typically require multi-session engagement over several weeks, and completion rates remain a concern, particularly in unguided programs.

An emerging body of evidence suggests that even a single session of CBT-I can produce meaningful improvement. Ellis and colleagues demonstrated that a single 60-minute “one-shot” CBT-I session with a self-help pamphlet produced a 60% remission rate at 1 month in individuals with acute insomnia, compared to 15% in the control group [16]. A recent review of single-session interventions for insomnia further supports their potential as early, accessible, and scalable tools within stepped-care frameworks [17]. In parallel, mass psychoeducation platforms—including public lectures, community workshops, and online media—have expanded the reach of health education beyond clinical settings, though their application to insomnia has been limited. These developments suggest an opportunity for single-session mass psychoeducation to address the insomnia treatment gap at two levels: as a preventive tool for the large population with subthreshold insomnia symptoms who are at risk of progression to chronic insomnia disorder, and as a first-step educational intervention for individuals with established insomnia who lack access to specialist care.

The present study introduces a novel theoretical framework for insomnia psychoeducation by incorporating Scarcity Theory [18]. Originally developed in behavioral economics, Scarcity Theory proposes that the experience of scarcity—whether of money, time, or sleep—creates a “tunneling” effect that narrows attentional focus and imposes a “bandwidth tax” on cognitive capacity, impairing judgment, planning, and self-regulation [19,20]. Although developed in the context of financial hardship, this framework has since been extended to health-relevant behaviors, such as dietary self-regulation, where tunneling and the bandwidth tax have been invoked to explain lapses in self-control [21]. The framework is also conceptually distinct from—though complementary to—the cognitive component of standard CBT-I: whereas conventional cognitive therapy for insomnia identifies and directly disputes individual dysfunctional beliefs [22], Scarcity Theory instead supplies a single unifying metaphor that reframes the worry–arousal cycle as a predictable consequence of depleted cognitive resources rather than targeting each belief in turn. We propose that this framework provides an intuitive and accessible lens through which individuals with insomnia can understand how sleep deprivation perpetuates the very cognitive patterns that maintain their insomnia: worry about sleep narrows cognitive bandwidth, which in turn amplifies dysfunctional beliefs and maladaptive behaviors, creating a self-reinforcing cycle. By making this mechanism visible, Scarcity Theory may facilitate the cognitive reappraisal that is central to CBT-I psychoeducation. The intervention combined this framework with self-distancing techniques drawn from psychological research [23], cognitive defusion concepts from Acceptance and Commitment Therapy (ACT) [24, 25], and principles of stimulus control therapy (SCT) [26] presented through a habit theory lens [27].

This study examined whether a single 60-minute mass psychoeducation session informed by Scarcity Theory and incorporating selected CBT-I psychoeducation and ACT concepts could reduce dysfunctional sleep beliefs and insomnia symptoms over a 4-week period. Based on the theoretical framework, we hypothesized that the intervention would produce significant reductions in dysfunctional beliefs about sleep (the Korean Dysfunctional Beliefs and Attitudes about Sleep-6, K-DBAS-6 [28]; primary outcome), insomnia symptom severity (the Insomnia Severity Index-3, ISI-3), and perceived cognitive burden of sleep deprivation (the Bandwidth Tax Visual Analogue Scale, BT-VAS) from baseline through the 4-week follow-up. We further explored whether the hypothesized causal sequence—from reduced perceived cognitive burden through belief change to symptom improvement—could be supported through mediation analysis, while recognizing that the study was likely underpowered for this test. To our knowledge, this is the first study to apply Scarcity Theory to insomnia psychoeducation and to examine the effects of a single-session mass psychoeducation intervention on dysfunctional sleep beliefs in an insomnia outpatient population.

## Materials and methods

### Study design and ethics

This was a prospective, single-arm, pre-post study with four assessment timepoints: baseline (T0, immediately before the lecture), immediate post-lecture (T1), 2-week follow-up (T2), and 4-week follow-up (T3). The study was conducted at Seoul National University Bundang Hospital in March 2026. The study protocol was approved by the Institutional Review Board of Seoul National University Bundang Hospital (B-2602-1026-305), and all participants provided written informed consent prior to enrollment.

### Participants

**Figure 1:**
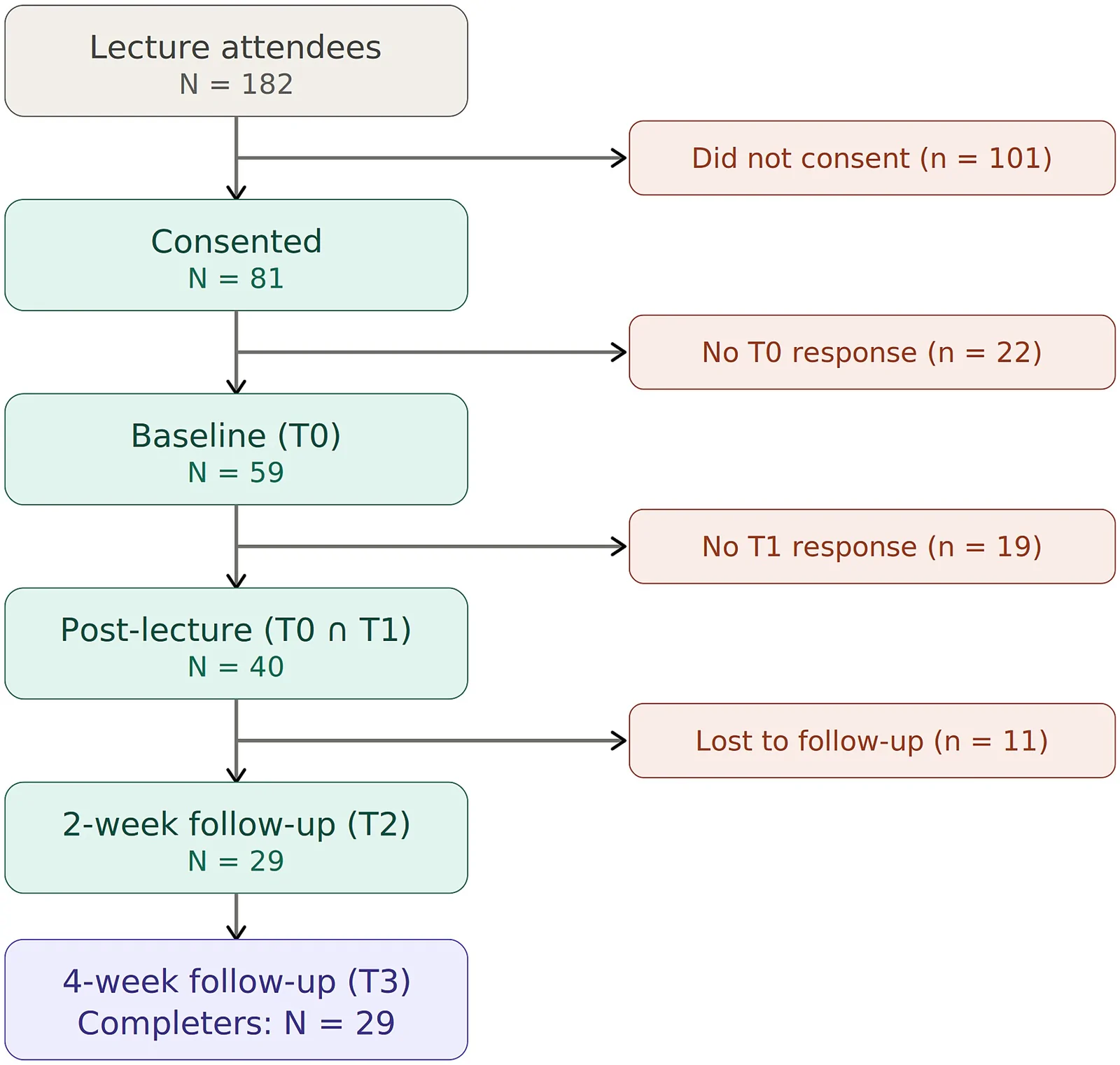
Participant flow diagram. A total of 182 individuals attended the public health lecture on insomnia. Of these, 81 provided written informed consent and 59 completed the baseline assessment (T0). Forty participants completed both the baseline and immediate post-lecture assessments (T0 and T1) and were included in the immediate effect analysis. Twenty-nine participants completed all four assessments (T0, T1, T2, and T3) and constituted the completer sample for longitudinal analyses. Reasons for non-completion at each stage included failure to provide consent, incomplete survey responses, and loss to follow-up. There were no significant differences in baseline characteristics between completers (*N* = 29) and non-completers (*N* = 30) (Table S1). T0, baseline (immediately pre-lecture); T1, immediately post-lecture; T2, 2-week follow-up; T3, 4-week follow-up.

Attendees of a public health lecture on insomnia were recruited through three channels: (1) text message invitations sent to patients who had visited the insomnia outpatient clinic within the preceding 2 years, distributed at 2 months, 4 weeks, and 2 weeks before the lecture and on the day of the lecture; (2) in-hospital posters; and (3) announcements on the hospital YouTube channel. Individual attendance pathways were not tracked; therefore, while the majority of attendees were presumed to be insomnia outpatients, participation by caregivers or members of the general public cannot be excluded. No specific diagnostic criteria for insomnia were applied for enrollment. Of 182 attendees, 81 provided written informed consent. Among these, 59 completed the baseline assessment (T0), 40 completed both the baseline and immediate post-lecture assessments (T0 and T1), and 29 completed all four assessments through the 4-week follow-up (completers). The participant flow is presented in Figure 1.

### Intervention

The intervention was a single 60-minute public psychoeducation lecture delivered by a board-certified neurologist (C.H.Y.), designed as a scalable, low-cost educational tool for individuals with insomnia complaints. The lecture was informed by Scarcity Theory [18], selected psychoeducational elements of CBT-I, and concepts from ACT [24,25]. Importantly, the intervention did not constitute a standard multi-session CBT-I program and did not include structured behavioral interventions, such as sleep restriction or systematic cognitive restructuring; rather, it introduced key psychoeducational concepts in a single-session format accessible to a large audience.

The lecture was structured in three phases. Phase 1*—*Problem recognition: Sleep regulation mechanisms, including the two-process model [29] and the 3P model of insomnia [30], were presented. The insomnia perpetuation cycle—encompassing hyperarousal [31] and conditioned arousal [32]—was reframed through the lens of Scarcity Theory, introducing the concept of a “bandwidth tax,” whereby sleep deprivation narrows cognitive capacity, amplifies worry, and impairs decision-making [18]. Phase 2—Cognitive reappraisal: Self-distancing techniques [23] and ACT-informed cognitive defusion concepts [24,25] were introduced to help participants observe and disengage from sleep-related ruminative thoughts rather than attempting to suppress them. Phase 3—Behavioral guidance: Principles of SCT [24] were introduced within a habit theory framework [27], providing participants with concrete behavioral strategies to restore the bed–sleep association.

The full lecture is publicly available online (https://www.youtube.com/watch?v=J5-p-9kRn-U).

### Outcome measures

The primary outcome was the Korean Dysfunctional Beliefs and Attitudes about Sleep-6 (K-DBAS-6) [28], a data-driven abbreviated version of the DBAS-16 [33] comprising items 4, 5, 7, 11, 13, and 15. Each item is rated on a 0–10 Likert scale, and the total score is calculated as the mean of the six items (range 0–10), consistent with DBAS-16 scoring conventions. The K-DBAS-6 was assessed at T0, T2, and T3.

Secondary outcomes included the following. The BT-VAS is a novel single-item measure derived from Scarcity Theory [18], on which participants rated the degree to which worry about sleep deprivation drains their everyday mental energy (0 = not at all; 5 = moderate; 10 = worry about sleep occupies the entire day, making it difficult to perform other tasks), assessed at all four timepoints (T0–T3). As a novel instrument, construct validity evidence is reported in the Results.

The ISI-3 is a 3-item abbreviated version of the Insomnia Severity Index [34] comprising the three symptom severity items (sleep-onset difficulty, sleep-maintenance difficulty, and early-morning awakening), each rated 0–4 (total range 0–12), assessed at T0, T2, and T3. This is a pragmatic abbreviation of the ISI for field administration in a mass setting, designed to minimize respondent burden while retaining the three core symptom severity items that directly assess the cardinal features of insomnia. This instrument has not been independently validated; construct validity evidence is presented in the Results.

The Patient Global Impression of Severity (PGI-S; range 1–7, where 1 = no symptoms, 2 = minimal, 3 = mild, 4 = moderate, 5 = slightly severe, 6 = severe, and 7 = extremely severe) was assessed at T0, T2, and T3. The Patient Global Impression of Improvement (PGI-I; range 1–7, where 1 = very much improved and 7 = very much worse) was assessed at T2 and T3 and served as the anchor for minimal clinically important difference (MCID) estimation.

Depression and anxiety were assessed at T0 and T3 with the Patient Health Questionnaire-2 (PHQ-2; range 0–6) [35] and Generalized Anxiety Disorder-2 (GAD-2; range 0–6) [36], respectively.

Stimulus control therapy adherence was assessed with four items evaluating key SCT principles: (1) going to bed only when sleepy, (2) refraining from using smartphones or watching television in bed, (3) getting out of bed immediately when unable to sleep, and (4) waking at a fixed time each morning (each item rated as 0 = never, 1 = rarely, 2 = sometimes, 3 = often, 4 = every day; total range 0–16), assessed at T2 and T3. At T1, participants identified their primary insight (“aha moment”) from the lecture by selecting one of four categories: scarcity and tunnel vision, psychological distancing, acceptance, or habit and automaticity.

Full item wording for the BT-VAS, ISI-3, SCT adherence scale, and “aha moment” is provided in Appendix 1.

### Procedures

After providing written informed consent (paper form), participants completed all assessments via online surveys accessed through QR codes. The baseline assessment (T0) was completed in the lecture hall immediately before the lecture, and the immediate post-lecture assessment (T1) was completed in the lecture hall immediately after the lecture. Follow-up assessments at 2 weeks (T2) and 4 weeks (T3) were administered via online surveys delivered through text message links.

### Statistical analysis

The immediate effect of the lecture on BT-VAS was evaluated using the Wilcoxon signed-rank test comparing T0 and T1 scores among all participants who completed both assessments (*N* = 40).

Longitudinal effects were evaluated using repeated measures analysis of variance (RM-ANOVA) with Bonferroni-corrected post-hoc pairwise comparisons across three timepoints (T0, T2, and T3) among completers (*N* = 29). RM-ANOVA was applied to the primary outcome (K-DBAS-6) and secondary longitudinal outcomes (ISI-3, BT-VAS, PGI-S). Pre-post changes in PHQ-2 and GAD-2 (T0 vs. T3) and SCT adherence (T2 vs. T3) were evaluated using Wilcoxon signed-rank tests. A complete-case approach was used for all longitudinal analyses; no imputation was performed. Sensitivity analyses using repeated measures analysis of covariance (RM-ANCOVA) adjusting for age and sex were conducted for K-DBAS-6, ISI-3, and BT-VAS. As a sensitivity analysis for attrition, linear mixed-effects models (LMM) with random intercepts for participants were fitted for K-DBAS-6, ISI-3, and BT-VAS, using all available observations from baseline respondents (*N* = 59) under a missing-at-random assumption. Time was entered as a categorical fixed effect (T0, T2, and T3). Overall time effects were tested using likelihood-ratio tests comparing models with and without the time term.

Exploratory subgroup analyses compared change scores (ΔK-DBAS-6, ΔISI-3) between groups defined by sleep medication use, prior CBT-I history, and baseline ISI-3 severity (median split) using Mann–Whitney U tests with rank-biserial *r*.

An exploratory mediation analysis tested the hypothesized causal pathway from immediate cognitive shift (ΔBT-VAS, T0 → T1) through belief change (ΔK-DBAS-6, T0 → T2) to symptom improvement (ΔISI-3, T0 → T3). Specifically, ΔK-DBAS-6 was examined as a mediator of the ΔBT-VAS–ΔISI-3 relationship. Indirect effects were estimated using bootstrapping with 5000 resamples and 95% bias-corrected confidence intervals. This analysis was recognized as likely underpowered; detection of a medium-sized indirect effect requires approximately *N* = 74 [37].

Anchor-based MCID was estimated using PGI-I at T3 as the external anchor. For the primary analysis, respondents were classified as improved (PGI-I ≤ 3) versus not improved (PGI-I ≥ 4), with a sensitivity analysis using a stricter threshold (PGI-I ≤2 vs. ≥3). The mean change in each outcome among participants reporting “minimally improved” (PGI-I = 3) was used as the MCID point estimate. Differences between responders and non-responders were tested using Mann–Whitney U tests with rank-biserial *r*.

For within-subject comparisons, effect sizes were reported as *d* z (mean difference divided by the standard deviation of difference scores) and *d* av (mean difference divided by the average of pre- and post-intervention standard deviations) for comparison with Cohen’s benchmarks (small = 0.2, medium = 0.5, and large = 0.8) [38].

All analyses were performed using R version 4.5.0 (R Foundation for Statistical Computing, Vienna, Austria). The significance level was set at α = .05 (two-tailed) for all tests.

## Results

### Participants

Baseline characteristics of the 29 completers are presented in Table 1. Participants were predominantly female (72.4%) with a mean age of 71.03 years (SD = 9.66; range 41–87). Nearly half (44.8%) reported current sleep medication use, and 31.0% had prior exposure to CBT-I. Baseline insomnia severity was moderate, with a mean PGI-S of 4.10 (SD = 1.05) and a mean ISI-3 of 6.34 (SD = 2.55); 72.4% (21/29) rated their insomnia severity as moderate or greater (PGI-S ≥ 4), confirming that the majority of completers experienced clinically meaningful insomnia at enrollment.

**Table 1:** Baseline demographics and clinical characteristics (*N* = 29)

Variable	Completers (*N* = 29)
Age, years, mean ± SD (range)	71.03 ± 9.66 (41–87)
Female sex, *n* (%)	21 (72.4)
Education, years, mean ± SD	14.28 ± 3.60
Current sleep medication use, *n* (%)	13 (44.8)
Occasional (<3×/week)	7 (24.1)
Regular (≥3×/week)	6 (20.7)
Prior CBT-I exposure, *n* (%)	9 (31.0)
**Baseline outcome measures**	
K-DBAS-6 (0–10), mean ± SD	4.52 ± 2.24
ISI-3 (0–12), mean ± SD	6.34 ± 2.55
BT-VAS (0–10), mean ± SD	5.38 ± 2.27
PGI-S [1–7], mean ± SD	4.10 ± 1.05
PHQ-2 (0–6), mean ± SD	1.79 ± 1.26
GAD-2 (0–6), mean ± SD	1.41 ± 1.21

K-DBAS-6, Korean Dysfunctional Beliefs and Attitudes about Sleep-6; ISI-3, Insomnia Severity Index-3; BT-VAS, Bandwidth Tax Visual Analogue Scale; PGI-S, Patient Global Impression of Severity; PHQ-2, Patient Health Questionnaire-2; GAD-2, Generalized Anxiety Disorder-2; CBT-I, cognitive-behavioral therapy for insomnia.

Occasional sleep medication use defined as <3 times per week; regular use ≥3 times per week.

Attrition from baseline to the 4-week follow-up was 50.8% (59–29). There were no significant differences in baseline demographics or outcome measures between completers and non-completers (Table S1). The full participant flow is detailed in Figure 1.

### Intervention effects

The lecture produced an immediate reduction in perceived cognitive burden. BT-VAS decreased from 5.50 (SD = 2.24) to 4.45 (SD = 2.24) immediately after the lecture (W = 39.5, p < .001, *d*z = 0.63, *d*av = 0.47; *N* = 40). This baseline value reflects the 40 participants in the immediate-effect analysis; the corresponding value among the 29 completers was 5.38 (Table 1).

**Figure 2:**
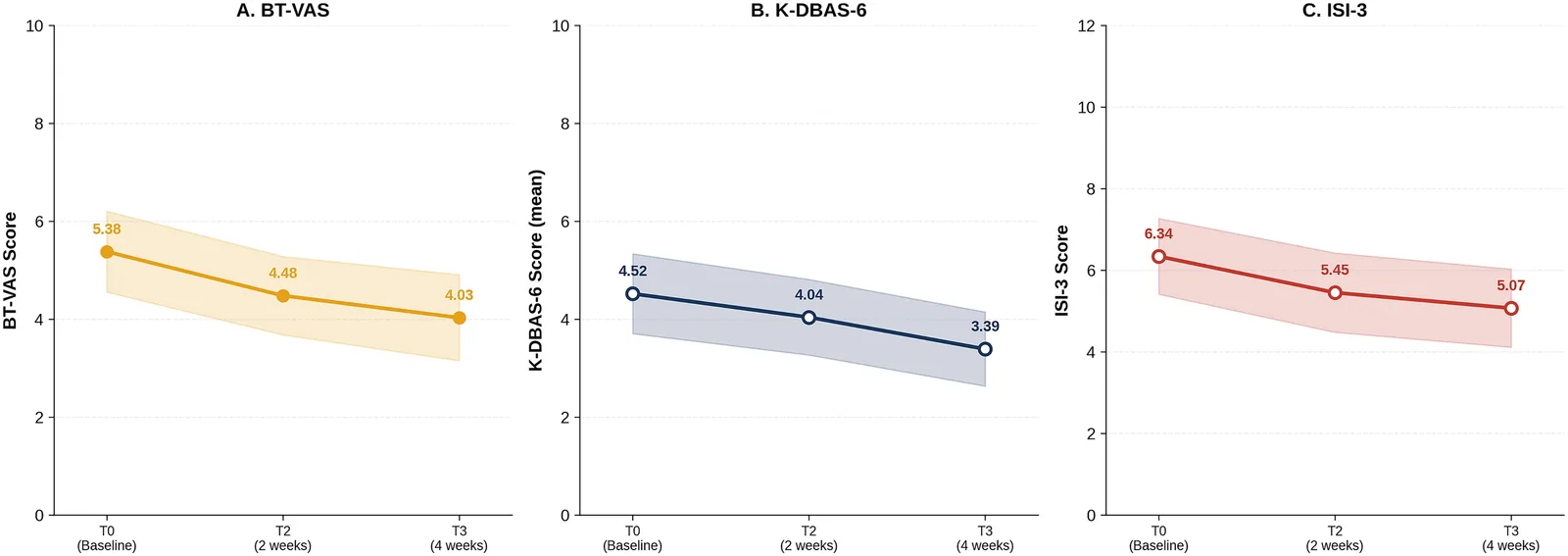
Trajectory of outcome measures across timepoints (N = 29). (A) Bandwidth Tax Visual Analogue Scale (BT-VAS). (B) Korean Dysfunctional Beliefs and Attitudes about Sleep-6 (K-DBAS-6). (C) Insomnia Severity Index-3 (ISI-3). Data points represent means; shaded bands represent 95% confidence intervals. Repeated measures analysis of variance with Bonferroni-corrected post-hoc comparisons.

The primary outcome, K-DBAS-6, showed a significant decrease over time (*F*(2,56) = 6.68, p = .003; Figure 2). Bonferroni-corrected post-hoc comparisons revealed a significant reduction from baseline to 4 weeks (4.52 ± 2.24 to 3.39 ± 2.08; p = .010, *d* z = 0.63, *d* av = 0.52), while the baseline-to-2-week change was not significant (p = .838). The 2-to-4-week comparison approached significance (p = .052), suggesting that belief change continued to accrue beyond the initial follow-up period. Item-level K-DBAS-6 changes are presented in Table S2; individual trajectories are shown in Figure S1.

Secondary longitudinal outcomes followed a consistent pattern of improvement (Figure 2). ISI-3 decreased significantly across timepoints (*F*(2,56) = 6.43, p = .003), with reductions evident at both 2 weeks (p = .038) and 4 weeks (p = .014, *d* z = 0.60, *d* av = 0.49). BT-VAS reduction was sustained through 4 weeks (*F*(2,56) = 7.22, p = .002, *d* z = 0.62, *d* av = 0.57). PGI-S showed the largest effect among all outcomes (*F*(2,56) = 14.91, p < .001, *d* z = 0.97, *d* av = 0.77). All longitudinal effects remained significant after adjusting for age and sex; neither covariate was significant (Table S3). LMMs including all 59 baseline respondents confirmed the robustness of these findings: overall time effects remained significant for K-DBAS-6 (LR χ^2^ = 11.73, p = .003), ISI-3 (LR χ^2^ = 14.41, p = .001), and BT-VAS (LR χ^2^ = 15.50, p < .001), with the pattern of pairwise differences consistent with the complete-case RM-ANOVA results (Table S3). Construct validity evidence for the ISI-3 and BT-VAS is provided in Table S4.

Depressive symptoms improved significantly from baseline to 4 weeks (PHQ-2: 1.79 ± 1.26 to 1.10 ± 1.05; p = .006, *d* z = 0.64, *d* av = 0.60). Anxiety showed a non-significant trend toward improvement (GAD-2: 1.41 ± 1.21 to 0.97 ± 1.09; p = .076).

SCT adherence did not change significantly between 2 and 4 weeks (9.10 ± 3.04 vs. 9.69 ± 3.93; p = .356), indicating that behavioral adherence was established by T2 and maintained. Among 40 participants who identified their primary insight at T1, the most frequently endorsed category was acceptance (52.5%), followed by psychological distancing (22.5%), scarcity and tunnel vision (15.0%), and habit and automaticity (10.0%).

### Clinical significance

At the 4-week follow-up, 72.4% of completers (21/29) reported global improvement (PGI-I ≤ 3). Mean changes across PGI-I categories showed a monotonic dose–response pattern, with greater improvement on all outcome measures among those reporting greater perceived benefit (Table 2A). Using the mean change in participants reporting “minimally improved” (PGI-I = 3, *n* = 12) as the MCID estimate, values were 1.32 for K-DBAS-6 (mean scale, 0–10), 1.58 for ISI-3, and 1.83 for BT-VAS. Responders (PGI-I ≤ 3) showed numerically greater change than non-responders on all outcomes, with medium effect sizes (rank-biserial *r* = 0.38–0.46), though differences did not reach statistical significance at the current sample size (Table 2B). A sensitivity analysis using a stricter responder threshold (PGI-I ≤ 2 vs. ≥3) showed smaller but directionally consistent effect sizes (rank-biserial *r* = 0.11–0.29; Table 2C).

**Table 2:** Clinical significance: MCID estimates and responder analysis (*N* = 29)

Panel A. Mean change (Δ) by PGI-I category at T3					
PGI-I	Label	*n*	ΔK-DBAS-6	ΔISI-3	ΔBT-VAS
1	Very much improved	1	−1.83	−4.00	−3.00
2	Much improved	8	−1.67	−1.75	−1.50
3	Minimally improved	12	−1.32	−1.58	−1.83
4	No change	7	−0.24	+0.29	+0.14
5	Minimally worse	1	−0.17	−2.00	−3.00
6	Much worse	0	–	–	–
7	Very much worse	0	–	–	–
**MCID estimate (PGI-I = 3)**			**−1.32**	**−1.58**	**−1.83**
**Panel B. Responder analysis: PGI-I ≤3 vs. ≥4**					
		***n***	**ΔK-DBAS-6**	**ΔISI-3**	**ΔBT-VAS**
Improved (≤3)		21	−1.48 ± 1.92	−1.76 ± 1.92	−1.76 ± 2.26
Not improved (≥4)		8	−0.23 ± 1.00	0.00 ± 2.20	−0.25 ± 1.58
p-value			.075	.058	.117
Rank-biserial *r*			0.44	0.46	0.38
**Panel C. Sensitivity: PGI-I ≤2 vs. ≥3**					
		***n***	**ΔK-DBAS-6**	**ΔISI-3**	**ΔBT-VAS**
Improved (≤2)		9	−1.69 ± 2.08	−2.00 ± 1.73	−1.67 ± 2.06
Not improved (≥3)		20	−0.88 ± 1.63	−0.95 ± 2.24	−1.20 ± 2.26
p-value			.229	.253	.665
Rank-biserial *r*			0.29	0.27	0.11

MCID, minimal clinically important difference; PGI-I, Patient Global Impression of Improvement (1 = very much improved to 7 = very much worse); K-DBAS-6, Korean Dysfunctional Beliefs and Attitudes about Sleep-6 (mean score, 0–10); ISI-3, Insomnia Severity Index-3 (0–12); BT-VAS, Bandwidth Tax Visual Analogue Scale (0–10). Panel A: Mean change (Δ) from T0 to T3. MCID is the mean change in the PGI-I = 3 (minimally improved) group. Panels B and C: Group differences tested using Mann–Whitney U tests. Effect size is rank-biserial *r*: small ≈ 0.10, medium ≈ 0.30, large ≈ 0.50. Values are mean ± SD.

### Exploratory analyses

The hypothesized mediation pathway—immediate cognitive shift (ΔBT-VAS) → belief change (ΔK-DBAS-6) → symptom improvement (ΔISI-3)—was not supported (Figure S2). The indirect effect was not significant (ab = −0.03, 95% bootstrap CI [−0.28 to 0.13]). Path a (ΔBT-VAS → ΔK-DBAS-6) was not significant (β = −0.40, p = .730), indicating that the immediate cognitive shift as measured by BT-VAS did not predict subsequent belief change. However, change in K-DBAS-6 and ISI-3 over the full 4-week study period were significantly correlated (*r* = 0.556, p = .002), consistent with the theoretical expectation that belief change and symptom improvement co-occur.

Exploratory subgroup analyses revealed no significant differences in ΔK-DBAS-6 or ΔISI-3 by sleep medication use, prior CBT-I history, or baseline ISI-3 severity (Table S5), suggesting that the intervention effects were broadly consistent across clinical subgroups. A trend toward greater ISI-3 improvement in participants with higher baseline severity was observed (rank-biserial *r* = 0.38, p = .080).

## Discussion

This study demonstrated that a single 60-minute mass psychoeducation session informed by Scarcity Theory, CBT-I psychoeducation principles, and ACT concepts produced significant, sustained reductions in dysfunctional sleep beliefs and insomnia symptoms over 4 weeks. The primary outcome, K-DBAS-6, showed a medium effect size (*d* z = 0.63) at the 4-week follow-up, with parallel improvements in ISI-3 (*d* z = 0.60), BT-VAS (*d* z = 0.62), and PGI-S (*d* z = 0.97). At 4 weeks, 72.4% of completers reported global improvement. These findings suggest that a scalable, single-session mass psychoeducation format can produce clinically meaningful changes in sleep-related cognition and symptoms in an insomnia outpatient population.

The magnitude of effects observed in this study is noteworthy given the minimal intensity of the intervention. The effect sizes for K-DBAS-6 and ISI-3 (*d* av = 0.49–0.52) fall within the medium range by conventional benchmarks and are comparable to those reported in meta-analyses of guided self-help and internet-delivered CBT-I programs that involve substantially greater time commitments [12, 15]. The single-session “one-shot” CBT-I trial by Ellis and colleagues, the closest precedent, achieved a 60% remission rate in acute insomnia [16]; our 72.4% responder rate (PGI-I ≤ 3) in a predominantly chronic insomnia population is encouraging, though direct comparison is limited by different outcome metrics—PGI-I ≤ 3 includes “minimally improved,” a less stringent criterion than ISI-based remission. Under a stricter threshold (PGI-I ≤ 2), 31.0% (9/29) reported substantial improvement. These findings suggest that mass psychoeducation can engage a meaningful proportion of participants with longstanding sleep difficulties.

An intriguing finding was the temporal pattern of K-DBAS-6 change: the baseline-to-2-week reduction was not statistically significant (p = .838), whereas the baseline-to-4-week reduction was (p = .010), and the 2-to-4-week change approached significance (p = .052). This progressive pattern suggests that cognitive change may require time to consolidate after initial psychoeducation. In contrast, ISI-3 showed significant improvement already at 2 weeks (p = .038), a dissociation that is consistent with the possibility that symptom improvement may be driven by behavioral changes (e.g. SCT adherence) that precede the full restructuring of dysfunctional beliefs. The finding that SCT adherence scores were already established at the 2-week assessment and maintained through 4 weeks is consistent with this interpretation.

The hypothesized mediation pathway—immediate cognitive shift (ΔBT-VAS) leading to belief change (ΔK-DBAS-6) leading to symptom improvement (ΔISI-3)—was not supported. The non-significant path from ΔBT-VAS to ΔK-DBAS-6 suggests that the BT-VAS, as a single-item novel measure, may not have captured the specific cognitive mechanism through which the intervention operates. Alternatively, the immediate post-lecture BT-VAS change may reflect a transient state shift rather than the initiation of a process that unfolds over weeks. Nonetheless, the significant concurrent correlation between ΔK-DBAS-6 and ΔISI-3 (*r* = 0.556, p = .002) is consistent with the broader theoretical expectation that belief change and symptom improvement co-occur, as observed in multi-session CBT-I research [10]. Among the aha moment responses, acceptance was endorsed most frequently (52.5%), followed by psychological distancing (22.5%), suggesting that the ACT-informed components resonated most strongly with participants.

A distinctive feature of this intervention was the incorporation of Scarcity Theory as a framing device for insomnia psychoeducation. Although this study cannot isolate the specific contribution of the Scarcity Theory framework from the other intervention components, we suggest that the bandwidth tax metaphor may have served a pedagogical function: providing participants with an intuitive, non-clinical language for understanding how sleep-related worry narrows cognitive capacity and perpetuates the insomnia cycle. This kind of explanatory metaphor may lower psychological resistance to the acceptance and defusion concepts introduced subsequently, by normalizing the cognitive consequences of sleep deprivation as a predictable resource-allocation phenomenon rather than a personal failing. The relatively high endorsement of acceptance as participants’ primary aha moment (52.5%) is consistent with—though not evidence for—this possibility. Future research using dismantling designs could evaluate whether the Scarcity Theory framing adds incremental benefit beyond standard CBT-I psychoeducation.

From a clinical perspective, these findings position single-session mass psychoeducation as a potential first step within a stepped-care model for insomnia [11]. The intervention requires no specialized training for the audience, can be delivered to large groups, and—critically—the lecture used in this study is publicly available on YouTube, enabling asynchronous, repeated viewing at no cost. The progressive pattern of K-DBAS-6 improvement between 2 and 4 weeks raises the possibility that some participants may have revisited the lecture material during the follow-up period, though this was not measured. For the substantial population with subthreshold insomnia symptoms who are at risk of progression to chronic insomnia disorder [3, 6], such low-barrier interventions could serve a preventive function. For individuals with established insomnia who lack access to specialist CBT-I, mass psychoeducation may provide foundational knowledge and behavioral tools that facilitate subsequent engagement with more structured treatments.

Regarding safety, the lecture explicitly included guidance against abrupt self-discontinuation of sleep medications and directed participants to consult their prescribing physicians before making any medication changes. The lecture also acknowledged populations for whom certain CBT-I components, particularly sleep restriction, may require caution, including individuals with bipolar disorder, epilepsy, or untreated obstructive sleep apnea. However, the mass delivery format inherently limits the ability to screen for contraindications or monitor individual responses, a consideration that should inform the design and deployment of future mass interventions.

Several limitations should be considered when interpreting these findings. First, the single-arm, pre-post design without a control group precludes causal attribution; observed improvements may reflect natural symptom fluctuation, regression to the mean, or placebo and expectancy effects inherent in attending a health lecture. Second, the sample was small (*N* = 29 completers), and attrition was substantial (50.8%). Although baseline characteristics did not differ significantly between completers and non-completers, compliance bias cannot be excluded: participants who perceived greater benefit from the lecture may have been more likely to complete follow-up assessments, potentially inflating the observed effect sizes. Conversely, the high rate of non-response may also reflect practical barriers to participation rather than lack of benefit. All surveys were administered online via QR codes and text links, and many attendees, predominantly older adults (mean age 71 years), required on-site assistance with QR code scanning and electronic survey completion. This digital literacy barrier may have contributed to both the initial gap between consent (*N* = 81) and baseline completion (*N* = 59) and the subsequent attrition during follow-up. Future studies involving older populations should consider alternative or hybrid data collection methods to minimize technology-related attrition. Third, the BT-VAS and ISI-3 are novel or self-abbreviated instruments that have not been independently validated; while we provided construct validity evidence, these findings require replication with established measures. In particular, the ISI-3 comprised only the three symptom-severity items of the ISI (sleep-onset, maintenance, and early-morning awakening) and omitted the items assessing sleep-related appraisal, distress, and daytime impairment [34, 39]. Because acceptance and psychological distancing were the insights most frequently endorsed by participants—processes that act specifically on the appraisal of, and distress about, sleep rather than on symptom frequency per se—the ISI-3 may have been relatively insensitive to a key domain of change. Administration of the full ISI might therefore have yielded a larger treatment effect, and the present estimates may be conservative in this respect. Fourth, the 4-week follow-up does not establish long-term durability of effects. Fifth, all outcomes were self-reported, with no objective sleep measurement. Sixth, the sample was predominantly older (mean age 71 years) and female (72.4%), recruited primarily through a hospital insomnia clinic, which limits generalizability to younger, community-based, or male-predominant populations. Seventh, individual attendance pathways were not tracked, the contribution of specific intervention components cannot be isolated, and no systematic adverse event monitoring was conducted in this mass delivery format. Additionally, the lecture was delivered by the principal investigator, and participants were recruited primarily from the outpatient clinic. Although the recruitment targeted all insomnia outpatients at the institution rather than the principal investigator’s own patients, the clinical setting and self-report outcome measures may have introduced demand characteristics and socially desirable responding. Finally, the study was conducted in a single cultural context (South Korea), and the applicability of the Scarcity Theory framing to other cultural settings remains to be examined.

Future studies should employ randomized controlled designs with appropriate comparators (e.g. sleep hygiene education, waitlist control), longer follow-up periods, objective sleep measures, and diverse populations. Dismantling studies could clarify the specific contribution of the Scarcity Theory framing. Tracking YouTube viewing behavior during follow-up periods would help to determine whether repeated engagement contributes to the observed progressive improvement in dysfunctional beliefs. Despite its limitations, this study provides preliminary evidence that a single 60-minute mass psychoeducation session informed by Scarcity Theory, CBT-I psychoeducation principles, and ACT concepts can produce significant, clinically meaningful reductions in dysfunctional sleep beliefs and insomnia symptoms. With medium effect sizes, a 72.4% responder rate, and a freely accessible delivery format, this approach warrants further investigation as a scalable, low-cost first-step intervention for insomnia.

## Supplementary Material

SLEEPADV_-_2026_-_102_Suppl_Accepted_zpag099

## Data Availability

The data underlying this article cannot be shared publicly due to participant privacy considerations under the Institutional Review Board approval. De-identified data may be made available to qualified researchers upon reasonable request to the corresponding author.
