# Supplementary material for: Breaking the sleep tunnel: effects of a single-session scarcity-informed mass psychoeducation on dysfunctional beliefs and insomnia symptoms
Source: Sleep Adv. 2026 Sep 3;7(3):zpag099. doi: 10.1093/sleepadvances/zpag099 (PMC13614423; doi:10.1093/sleepadvances/zpag099)
Supplement: Supplementary file 1 [file sleepadv_-_2026_-_102_suppl_accepted_zpag099.docx]

**Supplementary Materials**

**Title: Breaking the Sleep Tunnel: Effects of a Single-Session Scarcity-Informed Mass Psychoeducation on Dysfunctional Beliefs and Insomnia Symptoms**

Chang-Ho Yun, MD, PhD^1,^*, Saeil Nam, BS^1^, Hyeongsuk Ryu, PhD^1^, Sang-Min Paik, MD, MMSc^1^, Jae Rim Kim, MD, PhD^2^, Jee-Eun Yoon, MD, MMSc^3^

^1^Department of Neurology, Seoul National University Bundang Hospital and Seoul National University College of Medicine, Seongnam, Republic of Korea

^2^Department of Neurology, Chungnam National University Sejong Hospital, Chungnam National University School of Medicine, Sejong, Republic of Korea

^3^Department of Neurology, Soonchunhyang University Bucheon Hospital, Bucheon, Republic of Korea

***Corresponding Author:**

Chang-Ho Yun, MD, PhD

Department of Neurology, Seoul National University Bundang Hospital

82 Gumi-ro 173beon-gil, Bundang-gu, Seongnam-si, Gyeonggi-do 13620, Republic of Korea

Tel: +82-31-787-7469

E-mail: [ych333@gmail.com](mailto:ych333@gmail.com)

**SUPPLEMENTARY TABLES**

**Table S1. Baseline Comparison of Completers and Non-Completers**

| **Variable** | **Completers (n = 29)** | **Non-completers (n = 30)** | **p-value** |
| --- | --- | --- | --- |
| Age, years, mean ± SD | 71.03 ± 9.66 | 73.00 ± 5.60* | .575 |
| Female sex, n (%) | 21 (72.4) | 9 (81.8)* | .696^ᵃ^ |
| K-DBAS-6, mean ± SD | 4.52 ± 2.24 | 4.07 ± 2.26 | .617 |
| ISI-3, mean ± SD | 6.34 ± 2.55 | 6.60 ± 2.43 | .801 |
| BT-VAS, mean ± SD | 5.38 ± 2.27 | 5.57 ± 2.60 | .872 |
| PGI-S, mean ± SD | 4.10 ± 1.05 | 4.13 ± 1.22 | .807 |

*Sex and age for non-completers were collected at T1 and available for 11 of 30; baseline outcome measures reflect all 30. All comparisons by Mann-Whitney U test except where noted. ^ᵃ^Fisher exact test. No significant differences were observed between groups on any variable.

**Table S2. Item-Level Analysis**

| ***Panel A. K-DBAS-6 item-level changes (N = 29)*** | | | | | |
| --- | --- | --- | --- | --- | --- |
| **Item** | **T0** | **T2** | **T3** | **dz** | **p-value** |
| 4. Fear of losing control over sleep | 4.83 ± 2.66 | 3.83 ± 2.73 | 3.17 ± 2.79 | 0.67 | .001 |
| 5. Daytime interference after poor sleep | 5.24 ± 2.95 | 5.31 ± 3.15 | 4.31 ± 2.80 | 0.44 | .036 |
| 7. Mood disturbance attribution to sleep | 4.45 ± 2.81 | 3.83 ± 2.79 | 3.45 ± 2.63 | 0.41 | .033 |
| 11. Inability to manage consequences | 3.93 ± 2.59 | 3.34 ± 2.64 | 2.83 ± 2.38 | 0.39 | .054 |
| 13. Insomnia as chemical imbalance | 4.17 ± 2.52 | 4.07 ± 2.52 | 3.28 ± 2.27 | 0.35 | .060 |
| 15. Medication as the only solution | 4.48 ± 3.08 | 3.86 ± 3.19 | 3.28 ± 2.88 | 0.49 | .021 |
|  | | | | | |
| ***Panel B. SCT adherence item-level scores (N = 29)*** | | | | | |
| **Item** | **T2** | **T3** |  | **p-value** |  |
| 1. Going to bed only when sleepy | 2.31 ± 0.89 | 2.28 ± 1.19 |  | .808 |  |
| 2. No smartphone/TV in bed | 2.21 ± 1.26 | 2.45 ± 1.35 |  | .429 |  |
| 3. Getting out of bed if unable to sleep | 1.76 ± 1.24 | 2.07 ± 1.19 |  | .166 |  |
| 4. Fixed wake time | 2.83 ± 1.14 | 2.90 ± 1.08 |  | .723 |  |

Panel A: K-DBAS-6 individual items (each scored 0–10). Values are mean ± SD. Effect size (dz) and p-values are for T0→T3 change (Wilcoxon signed-rank test). Item numbers correspond to DBAS-16 item numbering. Panel B: SCT adherence items (each scored 0–4: 0 = never, 4 = every day). p-values are for T2→T3 change (Wilcoxon signed-rank test).

**Table S3. Sensitivity Analyses**

| ***Panel A. RM-ANCOVA adjusting for age and sex (N = 29)*** | | | | | | |
| --- | --- | --- | --- | --- | --- | --- |
| **Outcome** | **Time effect** |  | **Age** |  | **Sex** |  |
|  | *Wald χ²* | *p-value* | *β* | *p-value* | *β* | *p-value* |
| K-DBAS-6 | 13.40 | .001 | −0.06 | .787 | 4.08 | .416 |
| ISI-3 | 12.90 | .002 | 0.05 | .248 | 1.01 | .318 |
| BT-VAS | 14.40 | < .001 | 0.07 | .096 | 0.68 | .423 |
|  | | | | | | |
| ***Panel B. Linear mixed-effects models (N = 59 baseline respondents)*** | | | | | | |
| **Outcome** | **Overall time** |  | **T2 vs. T0** |  | **T3 vs. T0** |  |
|  | *LR χ²* | *p-value* | *β (SE)* | *p-value* | *β (SE)* | *p-value* |
| K-DBAS-6 | 11.73 | .003 | −0.41 (0.30) | .173 | −1.06 (0.30) | < .001 |
| ISI-3 | 14.41 | .001 | −0.94 (0.35) | .007 | −1.31 (0.35) | < .001 |
| BT-VAS | 15.50 | < .001 | −0.93 (0.35) | .008 | −1.38 (0.35) | < .001 |

Panel A: Repeated measures ANCOVA with age (continuous) and sex (binary) as covariates among completers (N = 29). Wald χ² tests the overall time effect (T0, T2, T3). β values represent unstandardized coefficients. Neither age nor sex was a significant covariate for any outcome.

Panel B: Linear mixed-effects models with random intercepts for participants, using all available observations from baseline respondents (N = 59; T0 = 59, T2 = 29, T3 = 29) under a missing-at-random assumption. LR χ² = likelihood-ratio chi-square test for the overall time effect (df = 2). β = unstandardized coefficient relative to T0; SE = standard error. All overall time effects remained significant, confirming the robustness of the complete-case RM-ANOVA results.

**Table S4. Construct Validity Evidence for ISI-3 and BT-VAS**

| ***Panel A. ISI-3 construct validity*** | | |
| --- | --- | --- |
| **Analysis** | **ρ / r** | **p-value** |
| ISI-3 × PGI-S (T0, N = 59) | .688 | < .001 |
| ISI-3 × PGI-S (T0, N = 29) | .767 | < .001 |
| ΔISI-3 × ΔPGI-S (T0→T3, N = 29) | .523 | .004 |
| ISI-3 × K-DBAS-6 (T0, discriminant, N = 59) | .610 | < .001 |
|  | | |
| ***Panel B. BT-VAS construct validity*** | | |
| **Analysis** | **ρ / r** | **p-value** |
| BT-VAS × K-DBAS-6 (T0, N = 59) | ρ = .561 | < .001 |
| BT-VAS × K-DBAS-6 (T0, N = 29) | ρ = .444 | .016 |
| ΔBT-VAS × ΔK-DBAS-6 (T0→T3, N = 29) | r = .434 | .019 |

Panel A: Convergent validity of ISI-3 with PGI-S (Spearman ρ). Discriminant validity with K-DBAS-6 (expected: moderate, as cognitive and symptom constructs overlap). Panel B: Convergent validity of BT-VAS with K-DBAS-6 (Spearman ρ for cross-sectional, Pearson r for change scores).

**Table S5. Exploratory Subgroup Analyses: Change Scores by Clinical Characteristics (N = 29)**

| **Subgroup** | **Outcome** | **G1, median [IQR]** | **G2, median [IQR]** | **p-value** | **Rank-biserial *r*** |
| --- | --- | --- | --- | --- | --- |
| ***Sleep medication*** | | | | | |
| Users (n = 13) vs. Non-users (n = 16) | |  | | | |
|  | ΔK-DBAS-6 | −0.67 [−2.50, 0.00] | −0.83 [−1.71, 0.17] | .792 | 0.06 |
|  | ΔISI-3 | −2.0 [−3.0, 1.0] | −1.0 [−3.0, 0.0] | .642 | 0.11 |
| ***CBT-I history*** | | | | | |
| Yes (n = 9) vs. No (n = 20) | |  | | | |
|  | ΔK-DBAS-6 | −0.50 [−1.67, −0.17] | −0.92 [−2.04, 0.08] | .795 | −0.07 |
|  | ΔISI-3 | −1.0 [−2.0, 0.0] | −2.0 [−3.0, 0.2] | .686 | −0.10 |
| ***ISI-3 baseline severity*** | | | | | |
| High (> 6, n = 13) vs. Low (≤ 6, n = 16) | |  | | | |
|  | ΔK-DBAS-6 | −1.00 [−1.83, −0.17] | −0.42 [−2.00, 0.54] | .392 | 0.19 |
|  | ΔISI-3 | −3.0 [−3.0, −2.0] | −1.0 [−2.0, 1.0] | .080 | 0.38 |

Values are median [interquartile range] of change scores (T0→T3). K-DBAS-6 reported on mean score scale (0–10). Group differences tested using Mann-Whitney U tests. Effect size is rank-biserial *r*. ISI-3 severity split at the sample median (> 6 vs. ≤ 6).

**SUPPLEMENTARY FIGURES**

**Figure S1. K-DBAS-6 individual trajectories (N = 29).**

Thin lines represent individual participant trajectories. Blue: responders (PGI-I ≤ 3, n = 21); red: non-responders (PGI-I ≥ 4, n = 8). Heavy line with circles: group mean. Values indicate group mean at each timepoint.

**Figure S2. Exploratory mediation model (N = 29).**

The hypothesized pathway from immediate cognitive shift (ΔBT-VAS, T0→T1) through belief change (ΔK-DBAS-6, T0→T2) to symptom improvement (ΔISI-3, T0→T3) was examined. Unstandardized path coefficients are shown with standard errors in parentheses. The indirect effect was not significant (ab = −0.03, 95% bootstrap CI [−0.28, 0.13]). Although the mediation pathway was not supported, concurrent change in K-DBAS-6 and ISI-3 over the full study period (T0→T3) was significantly correlated (r = .556, p = .002), consistent with the theoretical co-occurrence of belief change and symptom improvement. This exploratory analysis was likely underpowered (N = 29 vs. recommended N ≈ 74 for medium-effect mediation^1^).

**APPENDIX S1. Questionnaire Items**

**Bandwidth Tax Visual Analogue Scale (BT-VAS)**

Participants responded to a single item: “How much do you feel that worry about sleep deprivation is currently draining your everyday mental energy?”

Responses were recorded on an 11-point numerical rating scale: 0 = not at all; 5 = moderate; 10 = worry about sleep occupies the entire day, making it difficult to perform other tasks.

**Insomnia Severity Index-3 (ISI-3)**

Three items from the Insomnia Severity Index^2^, each rated 0–4 (0 = no problem; 4 = very severe):

(1) Difficulty falling asleep (sleep-onset insomnia)

(2) Difficulty staying asleep or difficulty returning to sleep after awakening (sleep-maintenance insomnia)

(3) Waking up too early and being unable to fall back asleep (early-morning awakening)

**Stimulus Control Therapy Adherence Scale (SCT; 4 items)**

Participants rated adherence to each behavior over the preceding 2 weeks on a 5-point scale: 0 = never; 1 = rarely; 2 = sometimes; 3 = often; 4 = every day.

(1) Going to bed only when sleepy

(2) Refraining from using smartphones or watching television in bed

(3) Getting out of bed immediately when unable to sleep

(4) Waking at a fixed time each morning

**Aha Moment Question (single-select)**

Administered immediately post-lecture (T1). Participants selected the single insight from the lecture that most changed their thinking:

(1) Scarcity and tunnel vision: “I realized that being trapped in the thought of sleep deprivation had narrowed my mental capacity.”

(2) Psychological distancing: “I came to see my anxious thoughts about sleep separately from myself, more objectively.”

(3) Acceptance: “Instead of forcing myself to sleep, I decided to accept my current state as it is.”

(4) Habit and automaticity: “I understood that insomnia is an automated response created by my habits.”

**REFERENCES**

1.	Fritz MS, Mackinnon DP. Required sample size to detect the mediated effect. Psychol Sci 2007;18:233-239.

2.	Morin CM, Belleville G, Belanger L, Ivers H. The Insomnia Severity Index: psychometric indicators to detect insomnia cases and evaluate treatment response. Sleep 2011;34:601-608.
